# *Pseudomonas aeruginosa* Activates PKC-Alpha to Invade Middle Ear Epithelial Cells

**DOI:** 10.3389/fmicb.2016.00255

**Published:** 2016-03-04

**Authors:** Rahul Mittal, M’hamed Grati, Denise Yan, Xue Z. Liu

**Affiliations:** ^1^Department of Otolaryngology, University of Miami Miller School of Medicine, MiamiFlorida, USA; ^2^Department of Biochemistry, University of Miami Miller School of Medicine, MiamiFL, USA; ^3^Department of Human Genetics, University of Miami Miller School of Medicine, MiamiFL, USA; ^4^Department of Otolaryngology, Xiangya Hospital, Central South UniversityChangsha, China

**Keywords:** chronic suppurative otitis media, *Pseudomonas aeruginosa*, PKC pathway, PepTag assay, MARCKs

## Abstract

Otitis media (OM) is a group of complex inflammatory disorders affecting the middle ear which can be acute or chronic. Chronic suppurative otitis media (CSOM) is a form of chronic OM characterized by tympanic membrane perforation and discharge. Despite the significant impact of CSOM on human population, it is still an understudied and unexplored research area. CSOM is a leading cause of hearing loss and life-threatening central nervous system complications. Bacterial exposure especially *Pseudomonas aeruginosa* is the most common cause of CSOM. Our previous studies have demonstrated that *P. aeruginosa* invades human middle ear epithelial cells (HMEECs). However, molecular mechanisms leading to bacterial invasion of HMEECs are not known. The aim of this study is to characterize the role of PKC pathway in the ability of *P. aeruginosa* to colonize HMEECs. We observed that otopathogenic *P. aeruginosa* activates the PKC pathway, specifically phosphorylation of PKC-alpha (PKC-α) in HMEECs. The ability of otopathogenic *P. aeruginosa* to phosphorylate PKC-α depends on bacterial OprF expression. The activation of PKC-α was associated with actin condensation. Blocking the PKC pathway attenuated the ability of bacteria to invade HMEECs and subsequent actin condensation. This study, for the first time, demonstrates that the host PKC-α pathway is involved in invasion of HMEECs by *P. aeruginosa* and subsequently to cause OM. Characterizing the role of the host signaling pathway in the pathogenesis of CSOM will provide novel avenues to design effective treatment modalities against the disease.

## Introduction

Otitis media (OM) refers to inflammation of the middle ear and mastoid cavity which can be acute or chronic (Minovi and Dazert, 2014; Qureishi et al., 2014). OM accounts for more than 25 million visits to physician’s offices annually and is associated with significant healthcare costs (Klein, 2000; Monasta et al., 2012). The global burden of disease study has attributed 4.68 million disability-adjusted life years (DALYs) to OM, a disease burden that is almost as high as the intestinal helminth infections (Murray et al., 2012). OM recurrence rates are high and acute OM (AOM) can progress to chronic OM (COM) despite appropriate treatment (Morris and Leach, 2009). One form of COM is chronic suppurative otitis media (CSOM) characterized by tympanic membrane perforation and purulent discharge (Verhoeff et al., 2006; Li MG et al., 2015). Children are at greater risk and suffer most frequently from CSOM, causing serious deterioration in their quality of life (Olatoke et al., 2008; Aarhus et al., 2015). CSOM remains an important global public health problem leading to hearing impairment, which may have serious long term effects on language, auditory, and cognitive development, as well as the educational progress of children (Elemraid et al., 2010; Taipale et al., 2011; Kolo et al., 2012; Jensen et al., 2013; Mittal et al., 2015). In addition, due to the proximity of ear to the brain, the spread of suppuration to the central nervous system (CNS) can lead to fatal extracranial and intracranial complications (Seven et al., 2005; Hossain et al., 2006; Dubey et al., 2010; Sun and Sun, 2014). Approximately 28,000 deaths are reported per year from CSOM due to CNS complications including brain abscess and meningitis (Acuin, 2004). Beside advances in medical therapy, CSOM still remains a clinically challenging disease. Despite the significant impact of CSOM on human population, the molecular mechanisms underlying the disease are still unknown. The emergence of antibiotic resistance and potential ototoxicity of antibiotics has created an immediate incentive to develop effective treatment modalities against CSOM. To design these therapeutic strategies, there is a need to understand the pathogenesis of CSOM. Bacterial infection of the middle ear is the most important factor that predisposes individuals to CSOM (Bluestone, 1998). *Pseudomonas aeruginosa* is the most common pathogen associated with CSOM (Saini et al., 2005; Yeo et al., 2007; Dayasena et al., 2011; Madana et al., 2011; Afolabi et al., 2012; Sattar et al., 2012). Our previous studies have demonstrated that *P. aeruginosa* invades human middle ear epithelial cells (HMEECs) and induces cytoskeletal rearrangements (Mittal et al., 2014). However, molecular mechanisms leading to actin condensation and invasion of HMEECs by *P. aeruginosa* are not known.

Protein kinase C (PKC) is a central host molecule that has been implicated in cytoskeletal reorganization (Brandt et al., 2002). A number of actin-binding proteins regulate the structure and dynamics of the actin cytoskeleton through organization of F-actin into a three-dimensional structure (dos Remedios et al., 2003; Paavilainen et al., 2004). Activities of these actin-binding proteins are controlled through various host signaling pathways to ensure proper spatial and temporal regulation of actin dynamics in cells (Khurana and George, 2008). One such signal transduction pathway that affects the actin cytoskeleton is the PKC pathway (Long and Freeley, 2014). PKC regulates the morphology of the F-actin cytoskeleton and thereby influences processes that are affected by remodeling of the microfilaments including cellular migration and neurite growth (Larsson, 2006; Quann et al., 2011; Michalczyk et al., 2013). PKC is composed of a family of phospholipid-dependent serine/threonine kinases mediating diverse cellular responses (Newton, 1995). In general, PKC has a catalytic domain that contains the ATP binding site and a regulatory domain containing the phospholipid and diacylglycerol (DAG) binding site (Luo and Weinstein, 1993; Poli et al., 2014).

Since PKC plays a central role in signaling events leading to changes in the cell membrane and cytoskeleton (Brandt et al., 2002), we hypothesized that PKC activation plays a crucial role in the invasion of HMEECs by *P. aeruginosa*. Our results showed that the PKC pathway is indeed involved in the ability of *P. aeruginosa* to colonize HMEECs and cause actin condensation. PKC inhibitors significantly blocked the invasion of HMEECs by otopathogenic *P. aeruginosa*. We also observed that *P. aeruginosa* of ear origin activates PKC during invasion of HMEECs for which bacterial OprF expression is necessary. The activated PKC translocates to the plasma membrane to initiate downstream signaling transduction events. To the best of our knowledge, this study for the first time demonstrates the role of PKC pathway in the pathogenesis of CSOM.

## Materials and Methods

### Cell Culture

Human middle ear epithelial cells (kindly provided by Dr. David Lim) were generated from human middle ear mucosa as described earlier (Mittal et al., 2014; Woo et al., 2015)^.^ HMEECs were cultured and maintained as described earlier (Lim and Moon, 2011; Mittal et al., 2014; Woo et al., 2014, 2015; Val et al., 2015). Briefly, HMEECs were cultured in a 1:1 mixture of Bronchial Epithelial Cell Basal Medium (Lonza, Allendale, NJ, USA) and Dulbecco’s Modified Eagle Medium (Cellgro, Manassas, VA, USA) supplemented with bronchial epithelial growth medium (BEGM) Singlequots (Lonza, Allendale, NJ, USA) and 10% fetal bovine serum (Life Technologies, Carlsbad, CA, USA). In some experiments, HMEECs were transfected with DN-PKC-α (Addgene Cambridge, MA, USA; Soh and Weinstein, 2003) using TransIT^®^-LT1 transfection reagent (Mirus, Madison, WI, USA) as per the manufacturer’s instructions. In separate experiments, HMEECs were treated with different concentrations of PKC inhibitors or actin polymerization or microtubule disrupting agents and then subjected to invasion assay.

### Bacterial Strains

A clinical otopathogenic strain of *P. aeruginosa* isolated from CSOM patient attending University of Miami Hospital is used in this study. The strain was identified and characterized as described previously (MacFaddin, 1976; Forbes et al., 1998; Saini et al., 2005; Yeo et al., 2007; Dayasena et al., 2011; Madana et al., 2011; Afolabi et al., 2012; Sattar et al., 2012). The isogenic OprF mutant (Δ*oprF*) and respective plasmid complemented strain (pOprF) consisting of the functional *oprF* gene was generated as described earlier (Woodruff and Hancock, 1989; Horton et al., 1990; Rietsch et al., 2005; Fito-Boncompte et al., 2011; Yakhnina et al., 2015). Bacteria were grown overnight in Luria broth at 37°C in a rotary shaker.

### Invasion Assays

Gentamicin protection assays were used to quantify the extent of bacterial invasion of HMEECs (Mittal et al., 2014). Briefly, HMEECs were infected with bacteria at various multiplicity of infection (MOI) and for different time-periods. After incubation, the cells were washed five times with warm RPMI followed by addition of medium containing gentamicin (200 μg/ml) and further incubated for 1 h at 37°C. The cells were washed three times with RPMI and then lysed with 1% saponin to release intracellular bacteria. Serial dilutions were then plated on blood agar plates and bacterial colonies were counted the next day. The binding of bacteria to HMEECs was determined by lysing the cells without adding gentamicin. To determine the effect of PKC inhibitors, HMEECs were pretreated with different concentrations of PKC inhibitory or control peptide, BIM I, Gö-6976, calphostin C, and chelerythrine for 30 min before infecting with bacteria and maintained in the medium for the entire infection period.

### PepTag Assay for Non-radioactive Detection of PKC Activity

The activation of PKC in HMEECs in response to *P. aeruginosa* infection was assessed by PepTag assay as per the manufacturer’s instructions (Promega, Madison, WI, USA). The assay uses brightly colored, fluorescent peptide substrates that are highly specific for the kinases in question. The hot pink color is imparted by the addition of a dye molecule to the PepTag Peptide substrate. Phosphorylation of PKC alters the peptide’s net charge from +1 to –1. This change in the net charge of the substrate allows the phosphorylated and non-phosphorylated versions of the substrate to be rapidly separated on an agarose gel. The phosphorylated species migrates toward the positive electrode, while the non-phosphorylated substrate migrates toward the negative electrode. HMEECs total cell lysates or membrane fractions (10–25 μg in 10 μl) were incubated with PKC reaction mixture at 33°C for 30 min. The reactions were stopped by placing the tubes in a boiling water bath. Samples were then run on agarose gel and bands were visualized under UV light.

### Quantitation of Extent of Phosphorylation by Spectrophotometry

Spectrophotometric method was used to quantitate PKC kinase activity as per manufacturer’s instructions. The phosphorylated bands at the negative electrode were excised by scalpel blade immediately after imaging the gel followed by heating at 95°C. The volume of the solution was adjusted to 250 μl with water. Samples were then mixed with gel solubilization buffer and glacial acetic acid followed by transferring to 96 well plates and reading the absorbance at 570 nm. Results were expressed in kinase units/ml.

### Western Blotting and Immunoprecipitation

Cells were infected with bacteria for different post-infection time periods, and lysates prepared. Protein concentrations of the extracts were measured using a BCA assay (Pierce, Rockford, IL, USA). Equivalent amounts of extracts were subjected to SDS-PAGE, transferred onto nitrocellulose membranes, and then blotted as described previously (Mittal and Prasadarao, 2010). For quantification, the Densitometric analysis was done using ImageJ software. For immunoprecipitation, cell lysates were incubated with anti-actin antibody overnight at 4°C followed by addition of Protein A+G sepharose beads. Immunoprecipitated beads were washed, resuspended in SDS sample buffer and resolved on SDS-PAGE. Immunoprecipitated proteins were transferred to nitrocellulose membrane and analyzed by Western blotting with anti- phospho-PKC-α antibody.

### Preparation of Membrane and Cytosolic Fractions

The membrane and cytosolic fractions were prepared from HMEECs using commercially available kit (Biovision, Milpitas, CA, USA) as per the manufacturer’s instructions.

### Immunofluorescence Microscopy

For staining of bacteria and actin, HMEECs were cultured in 8-well chamber slides and infected with *P. aeruginosa* for varying time periods. After incubation, cells were washed three times with PBS buffer and then fixed and permeabilized with BD cytofix and cytoperm reagent (BD Biosciences, San Jose, CA, USA) for 30 min. After washing, the cells were blocked with 3% normal goat serum (NGS) for 30 min and then incubated with anti-Phospho PKC-α antibody (Abcam, Cambridge, MA, USA) for 45 min followed by Alexa Fluor 488 antibody (Life Technologies, Carlsbad, CA, USA). After washing, cells were counterstained for actin with rhodamine phalloidin (Life Technologies, Carlsbad, CA, USA) for 45 min, washed and mounted in an antifade Vectashield solution containing 4′,6-diamidino-2-phenylindole (DAPI; Vector Laboratories, Burlingame, CA, USA). The cells were viewed with a Zeiss LSM 710 microscope (Carl Zeiss, Germany) and images were assembled using Adobe Photoshop 7.0.

### Statistical Analysis

Statistical significance was determined by a paired, two-tailed Student’s *t*-test using SPSS software. Values of *P* < 0.05 were considered to be statistically significant.

## Results

### PKC Inhibitors Attenuate Invasion of HMEECs by *P. aeruginosa*

**FIGURE 1:**
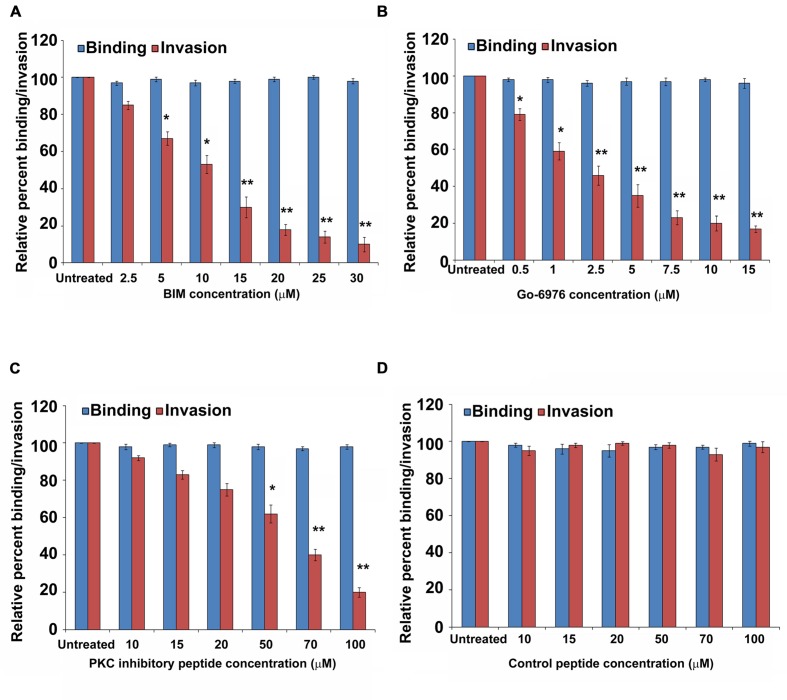
**PKC inhibitors prevent invasion of HMEECs by *P. aeruginosa*.** HMEECs were pretreated with different concentrations of BIM I **(A)**, Gö-6976 **(B)**, PKC inhibitory peptide **(C)**, or control peptide **(D)** for 30 min and then infected with *P. aeruginosa* at an MOI of 10 for 2 h. Bacterial adhesion and invasion was then determined. The results are expressed as percentage compared to the bacterial adhesion/invasion in untreated infected cells. Data represents mean ± SD and is representative of four individual experiments carried out in triplicate. ^∗^*P* < 0.01 or ^∗∗^*P* < 0.001 compared to control.

Our previous studies have demonstrated that *P. aeruginosa* can invade HMEECs and induce cytopathic effects (Mittal et al., 2014). To determine the role of PKC in cell invasion, HMEECs were infected with *P. aeruginosa* in the presence and absence of different concentrations of PKC inhibitors, bisindolylmaleimide I (BIM I), Gö-6976, PKC inhibitory peptide, calphostin C and chelerythrine. Bacterial cell invasion was then determined by gentamicin protection assay. We observed that with increase in concentrations of PKC inhibitors, there was significant decrease in invasion of HMEECs by *P. aeruginosa* (*P* < 0.01; **Figures 1A–C**). A concentration of 10 μM of BIM I was able to inhibit the invasion of *P. aeruginosa* by 50% whereas a dose of 30 μM was able to inhibit invasion by 90% (**Figure 1A**). Gö-6976 was able to block the invasion of *P. aeruginosa* by 85% at a concentration of 15 μM (**Figure 1B**). The myristoylated PKC inhibitory peptide at a concentration of 50 μM was able to block the invasion by 40% whereas a dose of 90 μM was able to inhibit the cell invasion by 80% (**Figure 1C**). However, control peptide had no effect on the invasion of *P. aeruginosa* into HMEECs at any of the tested concentrations (**Figure 1D**). Similar results of decreased cell invasion was observed with other PKC inhibitors, calphostin C and chelerythrine (Supplementary Figures S1 and S2). We did not observe any difference in the binding of *P. aeruginosa* to HMEECs in the presence of PKC inhibitors (**Figures 1A–C** and Supplementary Figures S1 and S2) indicating that the observed differences in invasion were not due to the differential bacterial binding. We also did not observe any toxic effects of these reagents on bacteria or on cells at the tested concentrations (data not shown). These results suggest that PKC plays a crucial role in colonization of HMEECs by *P. aeruginosa*.

### *P. aeruginosa* Activates PKC during HMEECs Invasion for Which OprF Expression is Required

**FIGURE 2:**
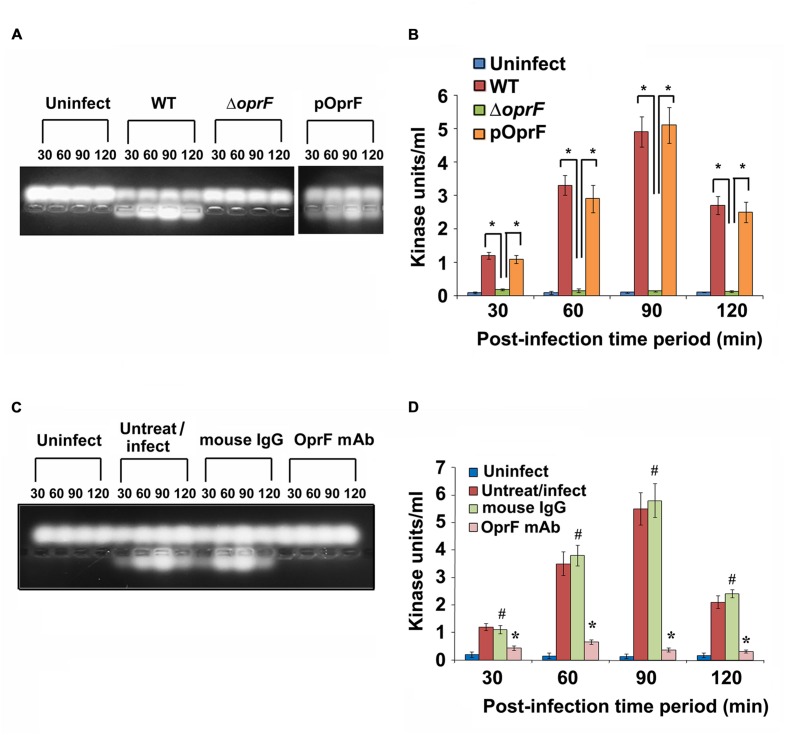
**Otopathogenic *P. aeruginosa* requires OprF to activate PKC in HMEECs.** Cells were infected with different strains of *P. aeruginosa* for varying time periods and subjected to non-radioactive PepTag assay **(A)**. PKC activity was quantified by excising the phosphorylated bands from the agarose gel and results were expressed in kinase units/ml **(B)**. In separate experiments, *P. aeruginosa* was pretreated with anti-OprF monoclonal antibody (mAb) or mouse IgG or left untreated and then used in the PKC assay **(C,D)**. Data represents mean ± SD. Results are representative of five independent experiments. ^#^*P* > 0.05 or ^∗^*P* < 0.001.

Since we observed that PKC plays a role in cell invasion, next we determined whether *P. aeruginosa* activates PKC in HMEECs. Our previous studies have demonstrated that bacterial OprF expression is required for the invasion of HMEECs by *P. aeruginosa* (Mittal et al., 2014). Therefore, we examined the ability of wild-type (WT) and Δ*oprF* mutant of *P. aeruginosa* to activate PKC in HMEECs by PepTag assay. Cells were infected with otopathogenic *P. aeruginosa* for varying time periods and PKC activation was determined using non-radioactive PepTag assay. HMEECs infected with WT *P. aeruginosa* demonstrated PKC activation within 30 min, which peaked at 90 min and then decreased at 120 min post-infection (**Figure 2A**). The increased PKC activity was approximately fourfold compared with PKC activity in control uninfected cells as estimated by the spectrophotometric method using phosphorylated substrate peptides (**Figure 2B**). In contrast, Δ*oprF* mutant induced significantly lower PKC activation than WT *P. aeruginosa* (^∗^*P* < 0.01). However, the cells infected with the complemented strain (pOprF strain) showed a similar pattern of PKC activation as the WT strain. These results suggest that interaction of OprF with HMEECs activates downstream signaling leading to cell invasion by *P. aeruginosa*.

To determine whether OprF has direct influence on the ability of *P. aeruginosa* to activate PKC, bacteria were pretreated with anti-OprF monoclonal antibody (mAb) and then used to infect HMEECs. Bacteria pretreated with isotype antibody or left untreated and uninfected served as the control group. We observed that anti-OprF monoclonal antibody treated bacteria failed to elicit PKC activation in HMEECs (**Figures 2C,D**). These results suggest that OprF directly contributes to the activation of PKC in HMEECs in response to *P. aeruginosa* infection.

### EGTA Treatment Abrogates *P. aeruginosa* Induced PKC Activation

**FIGURE 3:**
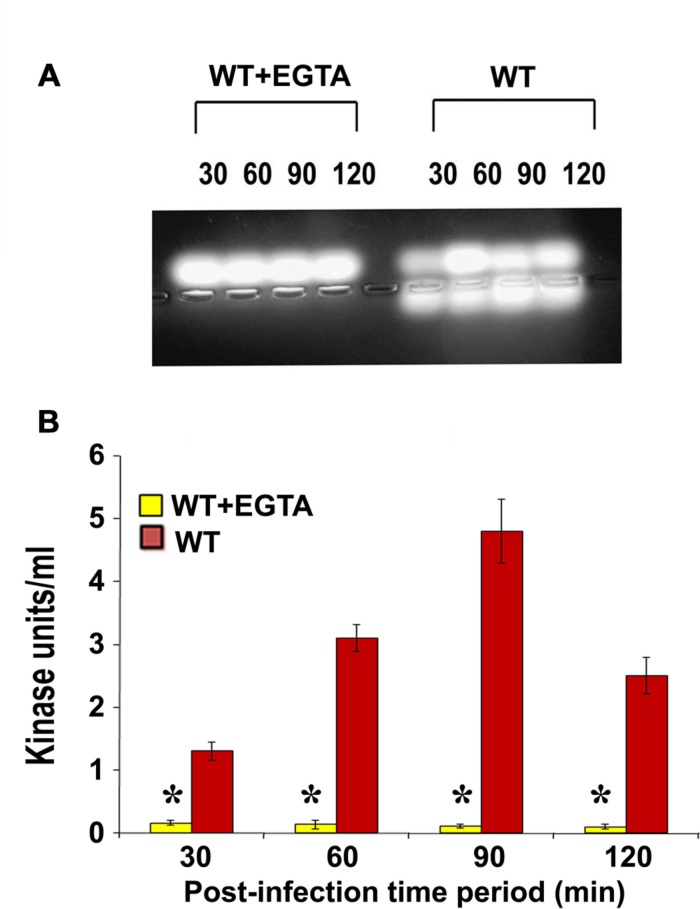
**EGTA abolishes *P. aeruginosa* induced PKC activation in HMEECs.** Cells were pretreated with EGTA for 30 min, and infected with *P. aeruginosa*. Total cell lysates were prepared and subjected to PepTag assay **(A)**. Quantitation of PKC activity by spectrophotometric assay demonstrated that EGTA prevents *P. aeruginosa* induced PKC activation in HMEECs **(B)**. Results are representative of three independent experiments. ^∗^*P* < 0.001.

Calcium plays an important role in the activation and regulation of conventional PKC isoforms which are most commonly implicated in cytoskeletal rearrangements (Herbert et al., 1990; Farah and Sossin, 2012). Therefore, we treated HMEECs with ethylene glycol tetraacetic acid (EGTA) to chelate calcium and infected with *P. aeruginosa*. Total cell lysates were then prepared and subjected to PepTag assay. We observed that PKC activation was completely abolished following pretreatment with EGTA (**Figure 3A**). Quantification of this data confirmed that EGTA significantly abrogated *P. aeruginosa* induced PKC activation in HMEECs compared to untreated cells (^∗^*P* < 0.001; **Figure 3B**). These findings suggest that the PKC activated in HMEECs following *P. aeruginosa* can be the conventional PKC isoform, PKC-α.

### Otopathogenic *P. aeruginosa* Specifically Activates PKC-α in HMEECs

**FIGURE 4:**
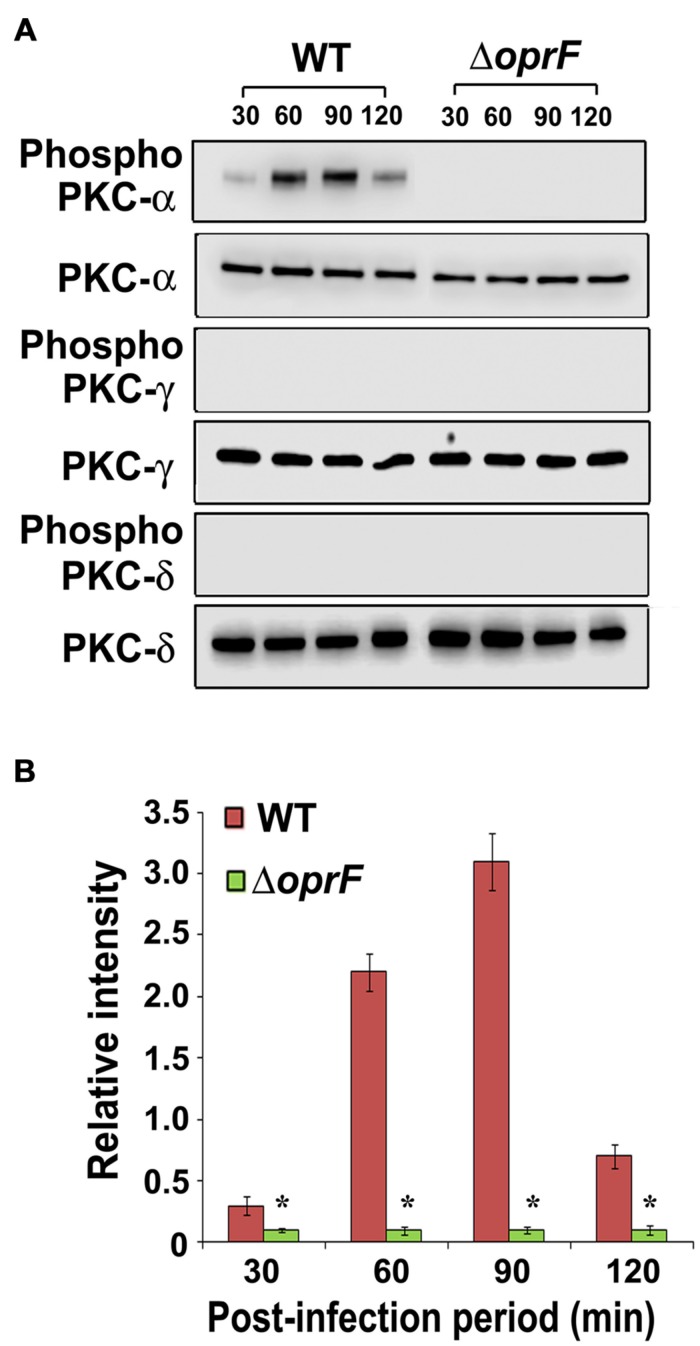
***P. aeruginosa* specifically activates PKC in HMEECs.** Cells were infected with *P. aeruginosa* for 30, 60, 90, and 120 min post-infection. Total cell lysates were prepared and subjected to Western blotting using either anti-phospho-PKC-α, anti-phospho-PKC-γ, or anti-phospho-PKC-δ antibodies **(A)**. The blots were stripped and reprobed with respective PKC antibodies to confirm equal loading of proteins **(A)**. The intensity of bands normalized to total PKC-α from two different blots was then calculated for quantification of phosphorylation of PKC-α **(B)**. Results are representative of three independent experiments. ^∗^*P* < 0.001.

Next we set forth to determine the PKC isotype activated by *P. aeruginosa* in HMEECs. PKC family is comprised of different isoforms that trigger distinct host signal transduction pathways (Newton, 1995). Cells were infected with WT, Δ*oprF* mutant or pOprF strains of *P. aeruginosa* for varying time-period, total cell lysates prepared and subjected to Western blotting. We observed that *P. aeruginosa* activated only PKC-α but not the other isoforms namely, PKC-γ and PKC-δ (**Figure 4A**). Time course study using anti-phospho-PKC-α specific antibodies revealed that *P. aeruginosa* phosphorylates PKC-α within 30 min post-infection, showing a peak at 90 min post-infection, and then decreases gradually afterward (**Figure 4B**). However, Δ*oprF* mutant failed to show any phosphorylation of PKC-α (**Figure 4A**). The ability of Δ*oprF* mutant to phosphorylate PKC was regained following complementation with pOprF. This data confirms our earlier PepTag assay data that phosphorylation of PKC-α in response to *P. aeruginosa* requires bacterial OprF expression.

### *Pseudomonas aeruginosa* Induces Translocation of PKC to the Membrane

**FIGURE 5:**
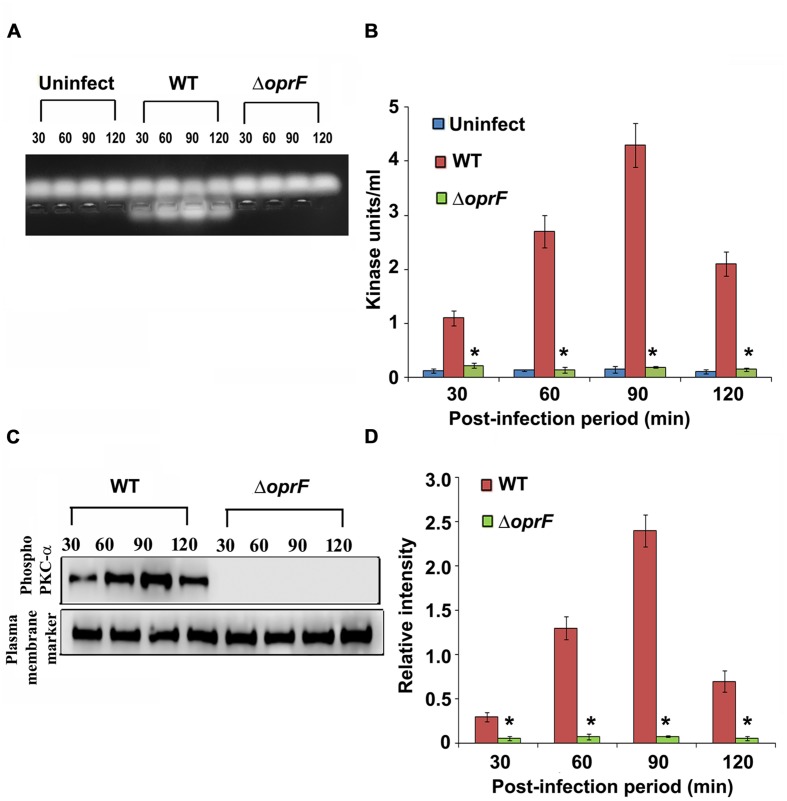
**Activated PKC localizes in the plasma membrane.** To determine the localization of activated PKC, membrane fractions were prepared from *P. aeruginosa* infected HMEECs and subjected to PepTag assay **(A)**. Quantitation of data showed that activated PKC resides in the membrane fraction **(B)**. In separate experiments, membrane fractions prepared from *P. aeruginosa* infected HMEECs were subjected to Western blotting and probed with anti-phospho-PKC-α antibody **(C)**. The blots were stripped and reprobed with anti-alpha 1 sodium potassium ATPase antibody (plasma membrane marker) to confirm equal loading of proteins. The intensity of bands normalized to plasma membrane marker was then calculated for quantification from two different blots **(D)**. Results are representative of three independent experiments. ^∗^*P* < 0.001.

The migration of phosphorylated PKC to the plasma membrane is essential in order to initiate downstream signaling (Newton, 1995). Therefore, we examined whether phosphorylated PKC in response to *P. aeruginosa* infection translocate to the plasma membrane of HMEECs. HMEECs were infected with *P. aeruginosa* and membrane fractions were prepared using commercially available kit (Biovision, Milpitas, CA, USA). PepTag assay revealed that phosphorylated PKC resides in the membrane (**Figure 5A**). We observed the same pattern of PKC activation in membrane fractions as we observed in total cell lysates. There was a fourfold increase in PKC activity at 90 min post-infection as compared to that at 30 min post-infection followed by a gradual decline in PKC activity at 120 min post-infection (**Figure 5B**).

To further confirm these results, we subjected the membrane fractions to Western blotting. We observed that in agreement with our PepTag assay, phosphorylated PKC-α was located in the membrane fraction (**Figure 5C**). There was an increase in phosphorylation of PKC-α with a corresponding increase in post-infection time period from 30 to 90 min as indicated by increase in band intensity followed by a gradual decline at 120 min post-infection (**Figure 5D**). Taken together, these results suggest that *P. aeruginosa* induced activated PKC translocate to the plasma membrane.

### Overexpression of Dominant Negative Form of PKC-α Inhibits PKC Activation and Invasion of HMEECs by *P. aeruginosa*

**FIGURE 6:**
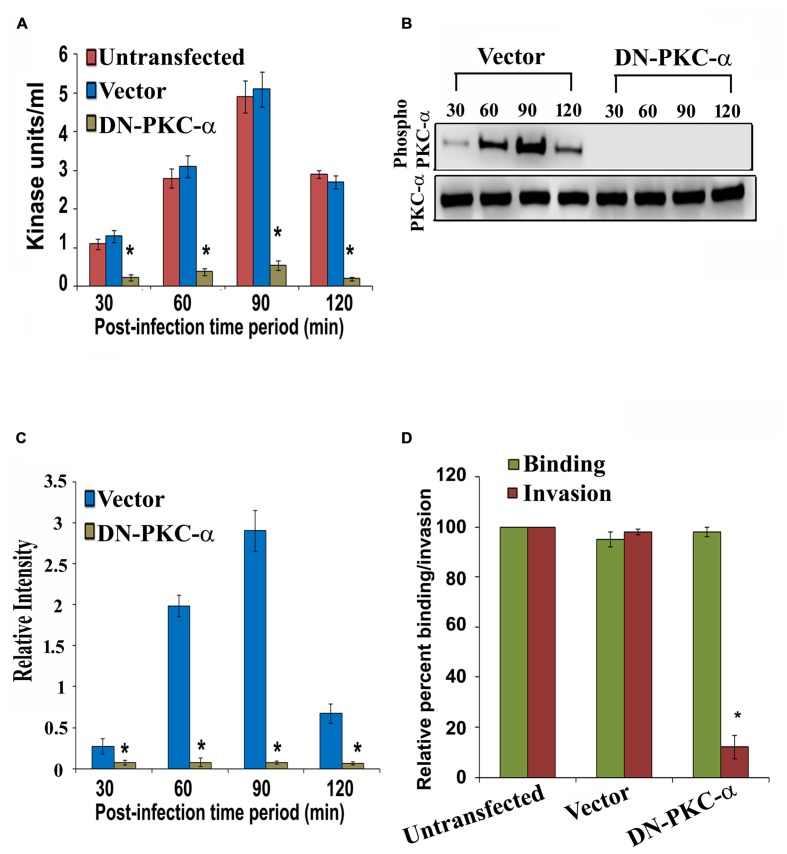
**Overexpression of dominant-negative (DN) PKC-α abolishes PKC activation and *P. aeruginosa* invasion.** HMEECs were transfected with DN PKC-α or vector alone or left untransfected, infected with *P. aeruginosa* and then subjected to PepTag assay. PKC kinase activity was then determined in DN PKC-α transfected cells or vector alone or untransfected HMEECs **(A)**. In separate experiments, total cell lysates from *P. aeruginosa* infected DN PKC-α or vector alone or left untransfected were subjected to Western blotting **(B)**. The extent of PKC-α phosphorylation was determined by densitometric analysis normalized to PKC-α from two different blots **(C)**. The invasion and adhesion of *P. aeruginosa* was also determined **(D)**. Results are representative of four independent experiments carried out in triplicate. ^∗^*P* < 0.001.

To characterize the role of PKC-α in cell invasion by *P. aeruginosa*, HMEECs were transfected with dominant negative (DN) PKC-α plasmid or vector alone or left untransfected. The DN PKC-α expression plasmid (PKC-α-cat/KR) encodes a truncated protein in which the N-terminal regulatory domain is deleted, while the catalytic domain (cat) containing amino acids 326–672 of PKC is preserved with a point mutation that abolishes the ATP binding ability (Soh and Weinstein, 2003). To determine whether expression of DN-PKC-α will prevent the activation of *P. aeruginosa* induced PKC, HMEECs lysates were subjected to PepTag assay. We observed that PKC activation was significantly attenuated in DN-PKC-α transfected cells compared to vector control or untransfected cells (**Figure 6A**). DN-PKC-α transfected cells showed fivefold decrease in PKC activation compared to vector control or untransfected cells (*P* < 0.001; **Figure 6A**). Similar results of decreased expression of PKC-α in DN-PKC-α transfected cells were observed with Western blotting (**Figures 6B,C**). In agreement with this data, the expression of DN-PKC-α into HMEECs significantly prevented the invasion of *P. aeruginosa* into HMEECs (**Figure 6D**). However, we did not observe any significant decrease in adhesion of *P. aeruginosa* to HMEECs (**Figure 6D**). This data demonstrates that colonization of HMEECs by *P. aeruginosa* requires active PKC-α.

### Phospho PKC-α Associates with Actin in *P. aeruginosa* Infected HMEECs

**FIGURE 7:**
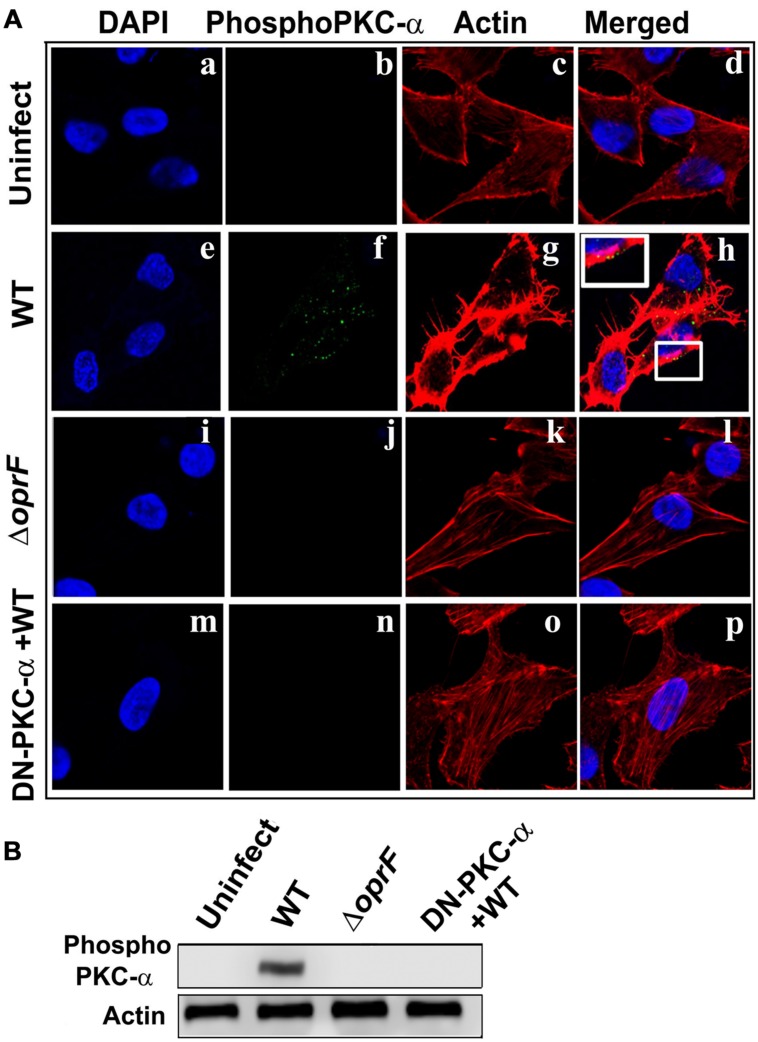
**Activated PKC colocalizes with the actin condensation.** HMEECs were left uninfected **(Aa–d)** or infected with WT **(Ae–h)** or Δ*oprF* mutant **(Ai–l)** *P. aeruginosa* and then stained with phospho-PKC-α antibody followed by Alex Fluor 488. Cells were counterstained with rhodamine-phalloidin to stain actin, DAPI to stain cell nuclei and subjected to confocal microscopy. In some experiments, HMEECs were transfected with DN-PKC-α **(Am–p)**, infected with WT *P. aeruginosa* and then subjected to confocal microscopy. In separate experiments, actin immunoprecipitates prepared from HMEECs were separated by SDS-PAGE and blotted with anti-phospho-PKC-α antibody **(B)**. Results are representative of three independent experiments.

In our previous studies, we observed that *P. aeruginosa* induces actin condensation during cell invasion (Mittal et al., 2014). Since PKC can play a central role in cytoskeletal rearrangements, we determined whether phospho-PKC-α colocalizes with actin bundles in *P. aeruginosa* infected HMEECs. Cells were infected with *P. aeruginosa* and stained with phospho-PKC-α antibody followed by secondary staining with Alexa Fluor 488. Cells were counterstained with rhodamine phalloidin to stain actin. Uninfected cells showed long actin filaments and no phospho-PKC-α staining (**Figures 7Aa–d**). In contrast, *P. aeruginosa* infected cells demonstrated actin accumulation beneath the bacterial binding sites that co-localizes with phospho-PKC-α staining at majority of sites as indicated by yellow color (**Figures 7Ae–h**). However, HMEECs infected with Δ*oprF* mutant failed to show actin condensation and phospho-PKC-α staining (**Figures 7Ai–l**). The overexpression of DN-PKC-α also prevented actin condensation and phosphorylation of PKC in agreement with our earlier data (**Figures 7Am–p**).

To confirm whether phospho-PKC-α associates with actin, we immunoprecipitated actin and then probed for phospho-PKC-α in *P. aeruginosa* infected HMEEC lysates. On par with our confocal microscopy results, we observed that phosphorylated PKC-α associates with actin in *P. aeruginosa* infected HMEECs (**Figure 7B**). However, no such association between actin and phospho-PKC-α was observed in Δ*oprF* mutant infected HMEECs or cells overexpressing DN-PKC-α. These results suggest that activated PKC-α in *P. aeruginosa* infected HMEECs interacts with actin cytoskeleton.

### Infection of HMEECs with *P. aeruginosa* Promotes Translocation of MARCKs from Plasma Membrane to Cell Cytosol

**FIGURE 8:**
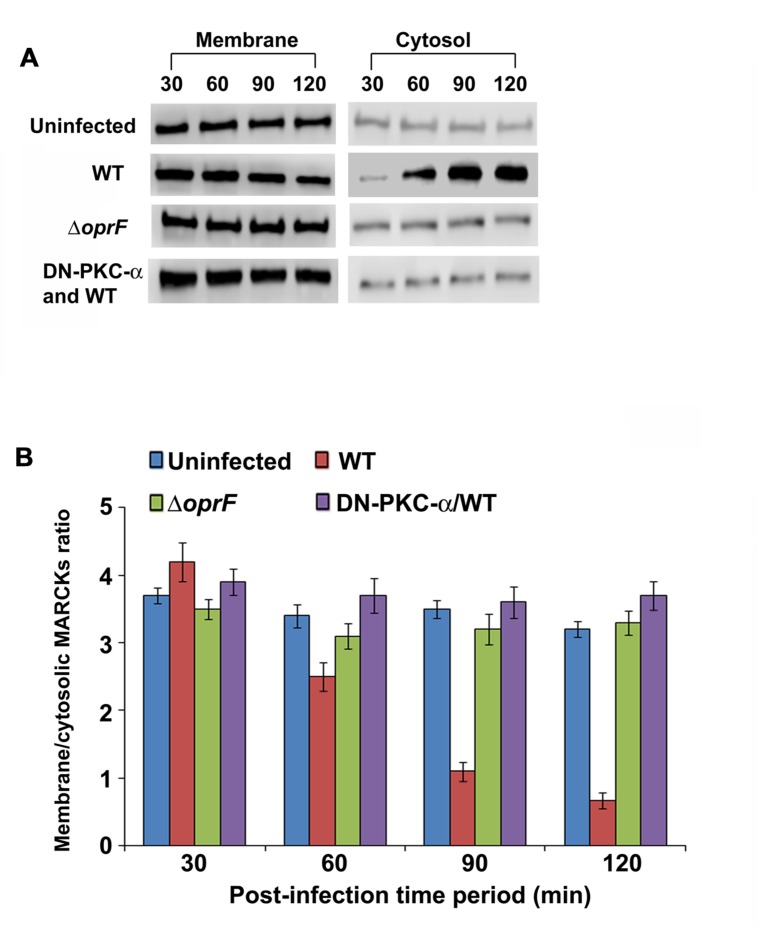
***P. aeruginosa* promotes translocation of PKC substrate, MARCKs, from membrane to cytosol in HMEECs.** Cells were left uninfected or infected with WT or OprF *P. aeruginosa* strains for varying time-periods, membrane and cytosolic fractions prepared and subjected to Western blotting with anti-MARCKs antibody **(A)**. In some experiments, HMEECs were transfected with DN-PKC-α before infecting with bacteria. The intensity of the bands was calculated by densitometric analysis and ratio of membrane associated to cytosolic MARCKs was calculated **(B)**. Results are representative of three independent experiments.

The myristoylated alanine-rich C kinase substrate (MARCKS) is one of the prominent substrates of PKC (Arbuzova et al., 2002). MARCKS normally resides in the plasma membrane where it plays a crucial role in the regulation of actin cytoskeleton (Arbuzova et al., 2002). Upon activation by PKC, MARCKs migrates from plasma membrane to the cytosol. Therefore we examined whether MARCKS translocate from plasma membrane to cytosol in HMEECs in response to *P. aeruginosa* infection. HMEECs were infected with *P. aeruginosa*, cytosolic and membrane fractions prepared and subjected to Western blotting. We observed that there was a decrease in MARCKs band intensity with increase in time period from 30 to 120 min post-infection in the membrane fraction of HMEECs infected with WT *P. aeruginosa* (**Figure 8A**). Quantification of this data confirmed that there was a 3.5-fold decrease in the presence of MARCKs in the membrane fraction at 120 min post-infection compared to at 30 min post-infection. On the other hand, MARCKS band intensity increased with increase in post-infection time period in the cytosolic fraction (**Figure 8A**). Densitometric analysis revealed a fivefold increase in MARCKs expression in the cytosol at 120 min post-infection compared to at 30 min post-infection. On the contrary, Δ*oprF* mutant showed no increase in the MARCKs expression in the cytosol or its decrease in the membrane fraction (**Figures 8A,B**). This data suggest that OprF expression on *P. aeruginosa* is involved in the translocation of MARCKs from membrane to cytosol. However, the expression of DN-PKC-α prevented the translocation of MARCKs from membrane to cytosol in WT *P. aeruginosa* infected cells suggesting that activation of PKC is necessary for this translocation (**Figures 8A,B**).

## Discussion

Chronic suppurative otitis media is the most prevalent disease worldwide, especially in developing countries, associated with potentially serious long term sequelae including hearing loss and fatal brain diseases. CSOM refers to insidious and chronic intractable inflammation of mucosa as well as submucosa with destruction of bone of the middle ear cleft characterized by persistent perforation of the tympanic membrane and recurrent otorrhea. The presence of bacteria in the middle ear and mastoid cavity is the most common cause of CSOM. However, the molecular mechanisms underlying CSOM are not known.

The invasion of bacteria into the host cells is considered a prerequisite to cause infection. Our previous studies have demonstrated that *P. aeruginosa* invades HMEECs in a dose and time dependent manner. However, molecular mechanisms that leads to colonization of HMEECs are not known. In this study, we demonstrated that PKC plays a central role in HMEECs invasion by *P. aeruginosa*. PKC specific inhibitors such as BIM I, Gö-6976, PKC inhibitory peptide, calphostin C and chelerythrine were able to block the bacterial cell invasion by more than 70–80%. BIM I is a cell permeable, very potent, specific, and selective inhibitor of PKC (Wanger et al., 2015). Gö-6976 is another PKC inhibitor that prevents the activation of calcium dependent isoforms in nanomolar concentrations while having no effect on the kinase activity of the calcium-independent PKC subtypes even at micromolar doses (Wang et al., 2015). PKC inhibitory peptide resembles the pseudosubstrate sequence of PKC-α whose function is to keep these kinases in their inactive state; thus, this peptide is a very specific competitive inhibitor of PKC-α (Eichholtz et al., 1993). Myristoylation of PKC inhibitory peptide makes it cell permeable and facilitates the entry inside cells. Calphostin C is a perylenequinone metabolite that targets classical and novel PKC isoforms and inhibits both phorbol ester binding and phosphotransferase activity of PKC through binding to the regulatory domain (Kobayashi et al., 1989; Xiao et al., 2015). Chelerythrine is a benzophenanthridine that is a potent and selective antagonist of classical as well as novel PKCs that targets the catalytic domain (Li SJ et al., 2015). Our results are in agreement with the findings of previous studies that showed that PKC inhibitors can prevent cell invasion by pathogenic microbes. The entry of a number of pathogenic viruses into host cells including the vesicular stomatitis virus, herpes simplex I virus, turkey herpes virus, vaccinia virus, Sindbis virus, human herpesvirus 8, and adenovirus type 2 has been shown to be blocked by PKC inhibitors (Constantinescu et al., 1991; Naranatt et al., 2003; Sieczkarski et al., 2003). BIM-I inhibits the entry of influenza virus inside Mink lung epithelial cells (Mv-1) during early infectious stages without affecting the viral binding as observed in the present study (Root et al., 2000). Earlier studies have also demonstrated that *Cryptosporidium* species utilizes PKC pathway to invade primary human and bovine intestinal cells (Hashim et al., 2006). PKC inhibitors significantly prevented the cell invasion by *Cryptosporidium* species. In agreement with our invasion data, we observed that *P. aeruginosa* activates PKC in HMEECs as early as 30 min post-infection, showing peak activation at 90 min post-infection. However, to the best of our knowledge, this study for the first time demonstrated the PKC activation for the invasion of HMEECs by otopathogenic bacteria.

PKCs are involved in a large variety of cell functions and signal transduction pathways regulating cell migration and polarity, proliferation, differentiation, and cell death (Newton, 1995). At least twelve different isoforms of PKC have been reported which have been categorized into three main types based on calcium dependency and activators: classical or conventional PKCs that are calcium dependent and DAG sensitive namely PKC-α, βI, βII, and γ; novel PKCs (δ, η, 𝜃, μ, ε, ξ) activated by DAG but not calcium; and atypical PKCs (μ, ζ, υ, ι) that are calcium independent and DAG insensitive (Newton, 1995). Each isotype triggers a different downstream signaling pathway that can determine the ultimate outcome of an infection. Therefore, there is a need to decipher the role of PKC during CSOM in order to understand the pathogenesis of the disease. In our study, we observed that PKC activation in HMEECs in response to *P. aeruginosa* is abrogated in the presence of calcium chelator, EGTA. This suggests that *P. aeruginosa* activates the calcium dependent classical isoform of PKC, most probably PKC-α. In agreement with this data, expression of DN PKC-α significantly prevented the invasion of HMEECs by otopathogenic *P. aeruginosa* and subsequent PKC activation. Western blotting demonstrated that otopathogenic *P. aeruginosa* specifically activates PKC-α but not the other isotypes. Interestingly, PKC-α activation has been correlated with increased cytokine production including TNF-α and IL-1β (Redig and Platanias, 2007). Therefore, it is possible that activation of PKC-α lead to exaggerated production of cytokines that is an important hallmark of CSOM. High levels of cytokines leads to tissue damage that further exacerbates the infection and worsens the complications associated with CSOM.

Outer membrane proteins (OMPs) play an important role in the interaction of pathogens with host cells (Galdiero et al., 2012; Confer and Ayalew, 2013). In this study, we observed that ability of otopathogenic *P. aeruginosa* to phosphorylate PKC-α depends on OprF expression. OprF is one of the most abundant OMPs/porin of *P. aeruginosa* (Sugawara et al., 2012). It has been demonstrated that OprF controls the production of the quorum-sensing-dependent virulence factors pyocyanin, elastase, lectin PA-1L, and exotoxin A as well as Type III secretion system associated enzymes, ExoS and ExoT, in non-otopathogenic strains of *P. aeruginosa* (Fito-Boncompte et al., 2011). However, our previous studies have demonstrated that OprF plays a direct role in interaction of otopathogenic *P. aeruginosa* with HMEECs (Mittal et al., 2014). The pretreatment of HMEECs with exogenous OprF or pretreatment of WT *P. aeruginosa* with anti-OprF mAb significantly reduced bacterial cell invasion. On par with these findings, in this study also, we observed that pretreatment of WT bacteria with OprF antibody abrogates *P. aeruginosa* induced PKC-α activation in HMEECs. These results suggest that OprF plays a unique role in otopathogenic *P. aeruginosa* and is directly involved in bacterial interaction with HMEECs.

The migration of activated PKC to the cell plasma membrane is essential to initiate the subsequent downstream signaling. In most cell types, PKCs are present in a primed, yet inactive, conformation in the cytosol, which are translocated to the membrane upon activation. PKC attains its active conformation when DAG recruits the inactive PKC to the membrane in a calcium-dependent manner, where membrane binding provides sufficient energy to disengage the pseudosubstrate, thus exposing the substrate binding site. We observed that activated PKC in response to *P. aeruginosa* migrates to the cell membrane. It is possible that translocation of phosphorylated PKC-α to the cell membrane facilitates the entry of *P. aeruginosa* inside HMEECs. A significant decrease in invasion of HMEECs by *P. aeruginosa* in the presence of PKC inhibitory peptide, which prevents the translocation of PKC to the membrane, further demonstrates the requirement of membrane-associated active PKC for the successful bacterial cell invasion. These results are consistent with the findings of other studies where activated PKC has been shown to translocate to the plasma membrane. The adhesion of *Leishmania donovani* to macrophages has been shown to produce rapid and transient redistribution of PKC from the cytosol to the plasma membrane (Bhunia et al., 1996). Enteropathogenic *E. coli* has also been shown to activate PKC in HeLa and T84 cells which then migrate to the plasma membrane (Crane and Oh, 1997).

Cytoskeletal rearrangements play an important role in entry and invasion of host cells by pathogens (Truong et al., 2014). In our earlier study, we showed that *P. aeruginosa* induces actin condensation during invasion of HMEECs. Here, we observed that *P. aeruginosa* utilizes PKC pathway to cause these cytoskeletal rearrangements. Confocal microscopy revealed that activated PKC-α colocalizes with the actin beneath the bacterial binding sites. In agreement with this findings, we observed the translocation of MARCKS from membrane to cytosol in *P. aeruginosa* infected HMEECs. MARCKs is a major PKC substrate that is associated with the plasma membrane and promotes cross linking of F-actin filaments in the dephosphorylated state (Blackshear, 1993). Phosphorylation of MARCKS by PKC facilitate its translocation from the plasma membrane to the cytosol leading to actin polymerization (Aderem, 1995). Thus, the translocation of MARCKs from membrane to cytosol leads to actin cytoskeletal rearrangement that facilitates the entry of *P. aeruginosa* into HMEECs.

In summary, our study provides novel insights into the pathogenesis of CSOM and decipher the role of PKC signaling pathway in the ability of *P. aeruginosa* to cause chronic ear infection. A comprehensive understanding of the role of PKC-α during CSOM employing animal models will pave the way to design effective treatment modalities against the disease and prevent consequent hearing loss as well as life-threatening CNS disorders.

## Author Contributions

RM, MG, DY, and XL conceived and designed the study. RM and MG performed the experiments and analyzed the data. RM, MG, DY, and XL wrote the manuscript. All the authors read and approved the final version of the manuscript.

## References

[B1] Aarhus L. Tambs K. Kvestad E. Engdahl B. (2015). Childhood otitis media: A cohort study with 30-year follow-up of hearing (The HUNT Study). *Ear Hear.* 36 302–308. 10.1097/AUD.0000000000000118 25401378 PMC4409918

[B2] Acuin J. (2004). *Chronic Suppurative Otitis Media - Burden of Illness and Management Options.* Geneva: World Health Organization http://www.who.int/pbd/publications/Chronic suppurativeotitis_media.pdf

[B3] Aderem A. (1995). The MARCKS family of protein kinase-C substrates. *Biochem. Soc. Trans.* 23 587–591. 10.1042/bst0230587 8566422

[B4] Afolabi O. A. Salaudeen A. G. Ologe F. E. Nwabuisi C. Nwawolo C. C. (2012). Pattern of bacterial isolates in the middle ear discharge of patients with chronic suppurative otitis media in a tertiary hospital in North central Nigeria. *Afr. Health Sci.* 12 362–367. 23382753 10.4314/ahs.v12i3.18 PMC3557674

[B5] Arbuzova A. Schmitz A. A. Vergères G. (2002). Cross-talk unfolded: MARCKS proteins. *Biochem. J.* 362 1–12. 10.1042/0264-6021:3620001 11829734 PMC1222354

[B6] Bhunia A. K. Sarkar D. Das P. K. (1996). *Leishmania donovani* attachment stimulates PKC-mediated oxidative events in bone marrow-derived macrophages. *J. Eukaryot. Microbiol.* 43 373–379. 10.1111/j.1550-7408.1996.tb05046.x 8822807

[B7] Blackshear P. J. (1993). The MARCKS family of cellular protein kinase C substrates. *J. Biol. Chem.* 268 1501–1504. 8420923

[B8] Bluestone C. D. (1998). Epidemiology and pathogenesis of chronic suppurative otitis media: implications for prevention and treatment. *Int. J. Pediatr. Otorhinolaryngol.* 42 207–223. 10.1016/S0165-5876(97)00147-X 9466224

[B9] Brandt D. Gimona M. Hillmann M. Haller H. Mischak H. (2002). Protein kinase C induces actin reorganization via a Src- and Rho-dependent pathway. *J. Biol. Chem.* 277 20903–20910. 10.1074/jbc.M200946200 11925438

[B10] Confer A. W. Ayalew S. (2013). The OmpA family of proteins: roles in bacterial pathogenesis and immunity. *Vet. Microbiol.* 163 207–222. 10.1016/j.vetmic.2012.08.019 22986056

[B11] Constantinescu S. N. Cernescu C. D. Popescu L. M. (1991). Effects of protein kinase C inhibitors on viral entry and infectivity. *FEBS Lett.* 292 31–33. 10.1016/0014-5793(91)80826-O 1659999

[B12] Crane J. K. Oh J. S. (1997). Activation of host cell protein kinase C by enteropathogenic *Escherichia coli*. *Infect. Immun.* 65 3277–3285. 9234787 10.1128/iai.65.8.3277-3285.1997 PMC175464

[B13] Dayasena R. Dayasiri M. Jayasuriya C. Perera D. (2011). Aetiological agents in chronic suppurative otitis media in Sri Lanka. *Australas. Med. J.* 4 101–104. 10.4066/AMJ.2011.549 23386888 PMC3562924

[B14] dos Remedios C. G. Chhabra D. Kekic M. Dedova I. V. Tsubakihara M. Berry D. A. (2003). Actin binding proteins: regulation of cytoskeletal microfilaments. *Physiol. Rev.* 83 433–473. 10.1152/physrev.00026.2002 12663865

[B15] Dubey S. P. Larawin V. Molumi C. P. (2010). Intracranial spread of chronic middle ear suppuration. *Am. J. Otolaryngol.* 31 73–77. 10.1016/j.amjoto.2008.10.001 20015716

[B16] Eichholtz T. de Bont D. B. de Widt J. Liskamp R. M. Ploegh H. L. (1993). A myristoylated pseudosubstrate peptide, a novel protein kinase C inhibitor. *J. Biol. Chem.* 268 1982–1986. 8420972

[B17] Elemraid M. A. Brabin B. J. Fraser W. D. Harper G. Faragher B. Atef Z. (2010). Characteristics of hearing impairment in Yemeni children with chronic suppurative otitis media: a case-control study. *Int. J. Pediatr. Otorhinolaryngol.* 74 283–286. 10.1016/j.ijporl.2009.12.004 20042241

[B18] Farah C. A. Sossin W. S. (2012). The role of C2 domains in PKC signaling. *Adv. Exp. Med. Biol.* 740 663–683. 10.1007/978-94-007-2888-2_29 22453964

[B19] Fito-Boncompte L. Chapalain A. Bouffartigues E. Chaker H. Lesouhaitier O. Gicquel G. (2011). Full virulence of *Pseudomonas aeruginosa* requires OprF. *Infect. Immun.* 79 1176–1186. 10.1128/IAI.00850-10 21189321 PMC3067511

[B20] Forbes B. A. Sahm D. F. Weissfeld A. S. (1998). *Bailey and Scott’s Diagnostic Microbiology*, 10th Edn. St. Louis, MI: Mosby Inc.

[B21] Galdiero S. Falanga A. Cantisani M. Tarallo R. Della Pepa M. E. D’Oriano V. (2012). Microbe-host interactions: structure and role of Gram-negative bacterial porins. *Curr. Protein Pept. Sci.* 13 843–854. 10.2174/138920312804871120 23305369 PMC3706956

[B22] Hashim A. Mulcahy G. Bourke B. Clyne M. (2006). Interaction of Cryptosporidium hominis and *Cryptosporidium parvum* with primary human and bovine intestinal cells. *Infect. Immun.* 74 99–107. 10.1128/IAI.74.1.99-107.2006 16368962 PMC1346631

[B23] Herbert J. M. Augereau J. M. Gleye J. Maffrand J. P. (1990). Chelerythrine is a potent and specific inhibitor of protein kinase C. *Biochem. Biophys. Res. Commun.* 172 993–999. 10.1016/0006-291X(90)91544-3 2244923

[B24] Horton R. M. Cai Z. L. Ho S. N. Pease L. R. (1990). Gene splicing by overlap extension: tailor-made genes using the polymerase chain reaction. *Biotechniques* 8 528–535. 2357375

[B25] Hossain M. M. Kundu S. C. Haque M. R. Shamsuzzaman A. K. Khan M. K. Halder K. K. (2006). Extracranial complications of chronic suppurative otitis media. *Mymensingh. Med. J.* 15 4–9. 16467754 10.3329/mmj.v15i1.24

[B26] Jensen R. G. Koch A. Homøe P. (2013). The risk of hearing loss in a population with a high prevalence of chronic suppurative otitis media. *Int. J. Pediatr. Otorhinolaryngol.* 77 1530–1535. 10.1016/j.ijporl.2013.06.025 23906989

[B27] Khurana S. George S. P. (2008). Regulation of cell structure and function by actin-binding proteins: villin’s perspective. *FEBS Lett.* 582 2128–2139. 10.1016/j.febslet.2008.02.040 18307996 PMC2680319

[B28] Klein J. O. (2000). The burden of otitis media. *Vaccine* 19 S2–S8. 10.1016/S0264-410X(00)00271-1 11163456

[B29] Kobayashi E. Nakano H. Morimoto M. Tamaoki T. (1989). Calphostin C (UCN-1028C), a novel microbial compound, is a highly potent and specific inhibitor of protein kinase C. *Biochem. Biophys. Res. Commun.* 159 548–553. 10.1016/0006-291X(89)90028-4 2467670

[B30] Kolo E. S. Salisu A. D. Yaro A. M. Nwaorgu O. G. (2012). Sensorineural hearing loss in patients with chronic suppurative otitis media. *Indian J. Otolaryngol. Head Neck Surg.* 64 59–62. 10.1007/s12070-011-0251-5 23449378 PMC3244579

[B31] Larsson C. (2006). Protein kinase C and the regulation of the actin cytoskeleton. *Cell. Signal.* 18 276–284. 10.1016/j.cellsig.2005.07.010 16109477

[B32] Li M. G. Hotez P. J. Vrabec J. T. Donovan D. T. (2015). Is chronic suppurative otitis media a neglected tropical disease? *PLoS Negl. Trop. Dis.* 9:e0003485 10.1371/journal.pntd.0003485 PMC4374690 25811602

[B33] Li S. J. Cui S. Y. Zhang X. Q. Yu B. Sheng Z. F. Huang Y. L. (2015). PKC in rat dorsal raphe nucleus plays a key role in sleep-wake regulation. *Prog. Neuropsychopharmacol. Biol. Psychiatry* 63 47–53. 10.1016/j.pnpbp.2015.05.005 25970525

[B34] Lim D. J. Moon S. K. (2011). Establishment of cell lines from the human middle and inner ear epithelial cells. *Adv. Exp. Med. Biol.* 720 15–25. 10.1007/978-1-4614-0254-1_2 21901615

[B35] Long A. Freeley M. (2014). Protein kinase C: a regulator of cytoskeleton remodelling and T-cell migration. *Biochem. Soc. Trans.* 42 1490–1497. 10.1042/BST20140204 25399559

[B36] Luo J. H. Weinstein I. B. (1993). Calcium-dependent activation of protein kinase C. The role of the C_2_ domain in divalent cation selectivity. *J. Biol. Chem.* 268 23580–23584. 8226885

[B37] MacFaddin J. (1976). *Biochemical Tests for Identification of Medical Bacteria*, 3rd Edn Philadelphia: Lippincott Williams and Wilkins.

[B38] Madana J. Yolmo D. Kalaiarasi R. Gopalakrishnan S. Sujatha S. (2011). Microbiological profile with antibiotic sensitivity pattern of cholesteatomatous chronic suppurative otitis media among children. *Int. J. Pediatr. Otorhinolaryngol.* 75 1104–1108. 10.1016/j.ijporl.2011.05.025 21715027

[B39] Michalczyk I. Sikorski A. F. Kotula L. Junghans R. P. Dubielecka P. M. (2013). The emerging role of protein kinase C 𝜃 in cytoskeletal signaling. *J. Leukoc. Biol.* 93 319–327. 10.1189/jlb.0812371 23192428 PMC3579025

[B40] Minovi A. Dazert S. (2014). Diseases of the middle ear in childhood. *GMS. Curr. Top. Otorhinolaryngol. Head Neck Surg.* 13:Doc11 10.3205/cto000114 PMC4273172 25587371

[B41] Mittal R. Grati M. Gerring R. Blackwelder P. Yan D. Li J. D. (2014). In vitro interaction of *Pseudomonas aeruginosa* with human middle ear epithelial cells. *PLoS One* 9:e91885 10.1371/journal.pone.0091885 PMC3954863 24632826

[B42] Mittal R. Lisi C. V. Gerring R. Mittal J. Mathee K. Narasimhan G. (2015). Current concepts in the pathogenesis and treatment of chronic suppurative otitis media. *J. Med. Microbiol.* 64 1103–1116. 10.1099/jmm.0.000155 26248613 PMC4835974

[B43] Mittal R. Prasadarao N. V. (2010). Nitric oxide/cGMP signalling induces *Escherichia coli* K1 receptor expression and modulates the permeability in human brain endothelial cell monolayers during invasion. *Cell. Microbiol.* 12 67–83. 10.1111/j.1462-5822.2009.01379.x 19732056 PMC2804784

[B44] Monasta L. Ronfani L. Marchetti F. Montico M. Vecchi Brumatti L. Bavcar A. (2012). Burden of disease caused by otitis media: systematic review and global estimates. *PLoS ONE* 7:e36226 10.1371/journal.pone.0036226 PMC3340347 22558393

[B45] Morris P. S. Leach A. J. (2009). Acute and chronic otitis media. *Pediatr. Clin. North Am.* 56 1383–1399. 10.1016/j.pcl.2009.09.007 19962027 PMC7111681

[B46] Murray C. J. Vos T. Lozano R. Naghavi M. Flaxman A. D. Michaud C. (2012). Disability-adjusted life years (DALYs) for 291 diseases and injuries in 21 regions, 1990–2010: a systematic analysis for the Global Burden of Disease Study 2010. *Lancet* 380 2197–2223. 10.1016/S0140-6736(12)61689-4 23245608

[B47] Naranatt P. P. Akula S. M. Zien C. A. Krishnan H. H. Chandran B. (2003). Kaposi’s sarcoma-associated herpesvirus induces the phosphatidylinositol 3-kinase-PKC–MEK-ERK signaling pathway in target cells early during infection: implications for infectivity. *J. Virol.* 77 1524–1539. 10.1128/JVI.77.2.1524-1539.2003 12502866 PMC140802

[B48] Newton A. C. (1995). Protein kinase C: structure, function, and regulation. *J. Biol. Chem.* 270 28495–28498. 10.1074/jbc.270.48.28495 7499357

[B49] Olatoke F. Ologe F. E. Nwawolo C. C. Saka M. J. (2008). The prevalence of hearing loss among schoolchildren with chronic suppurative otitis media in Nigeria, and its effect on academic performance. *Ear. Nose Throat. J.* 87 E19. 19105130

[B50] Paavilainen V. O. Bertling E. Falck S. Lappalainen P. (2004). Regulation of cytoskeletal dynamics by actin-monomer-binding proteins. *Trends Cell. Biol.* 14 386–394. 10.1016/j.tcb.2004.05.002 15246432

[B51] Poli A. Mongiorgi S. Cocco L. Follo M. Y. (2014). Protein kinase C involvement in cell cycle modulation. *Biochem. Soc. Trans.* 42 1471–1476. 10.1042/BST20140128 25233434

[B52] Quann E. J. Liu X. Altan-Bonnet G. Huse M. (2011). A cascade of protein kinase C isozymes promotes cytoskeletal polarization in T cells. *Nat. Immunol.* 12 647–654. 10.1038/ni.2033 21602810 PMC3119370

[B53] Qureishi A. Lee Y. Belfield K. Birchall J. P. Daniel M. (2014). Update on otitis media - prevention and treatment. *Infect. Drug Resist.* 7 15–24. 10.2147/IDR.S39637 24453496 PMC3894142

[B54] Redig A. J. Platanias L. C. (2007). The protein kinase C (PKC) family of proteins in cytokine signaling in hematopoiesis. *J. Interferon Cytokine Res.* 27 623–636. 10.1089/jir.2007.0007 17784814

[B55] Rietsch A. Vallet-Gely I. Dove S. L. Mekalanos J. J. (2005). ExsE, a secreted regulator of type III secretion genes in *Pseudomonas aeruginosa*. *Proc. Natl. Acad. Sci. U.S.A.* 102 8006–8011. 10.1073/pnas.0503005102 15911752 PMC1142391

[B56] Root C. N. Wills E. G. McNair L. L. Whittaker G. R. (2000). Entry of influenza viruses into cells is inhibited by a highly specific protein kinase C inhibitor. *J. Gen. Virol.* 2000 2697–2705. 10.1099/0022-1317-81-11-2697 11038382

[B57] Saini S. Gupta N. Aparna Seema Sachdeva O. P. (2005). Bacteriological study of paediatric and adult chronic suppurative otitis media. *Indian J. Pathol. Microbiol.* 48 413–416. 16761774

[B58] Sattar A. Alamgir A. Hussain Z. Sarfraz S. Nasir J. Badar-e-Alam (2012). Bacterial spectrum and their sensitivity pattern in patients of chronic suppurative otitis media. *J. Coll. Physicians Surg. Pak.* 22 128–129. 22313657

[B59] Seven H. Coskun B. U. Calis A. B. Sayin I. Turgut S. (2005). Intracranial abscesses associated with chronic suppurative otitis media. *Eur. Arch. Otorhinolaryngol.* 262 847–851. 10.1007/s00405-004-0903-0 15959795

[B60] Sieczkarski S. B. Brown H. A. Whittaker G. R. (2003). Role of protein kinase C betaII in influenza virus entry via late endosomes. *J. Virol.* 77 460–469. 10.1128/JVI.77.1.460-469.2003 12477851 PMC140583

[B61] Soh J. W. Weinstein I. B. (2003). Roles of specific isoforms of protein kinase C in the transcriptional control of cyclin D1 and related genes. *J. Biol. Chem.* 278 34709–34716. 10.1074/jbc.M302016200 12794082

[B62] Sugawara E. Nagano K. Nikaido H. (2012). Alternative folding pathways of the major porin OprF of *Pseudomonas aeruginosa*. *FEBS J.* 279 910–918. 10.1111/j.1742-4658.2012.08481.x 22240095 PMC3338866

[B63] Sun J. Sun J. (2014). Intracranial complications of chronic otitis media. *Eur. Arch. Otorhinolaryngol.* 271 2923–2926. 10.1007/s00405-013-2778-4 24162767

[B64] Taipale A. Pelkonen T. Taipale M. Bernardino L. Peltola H. Pitkäranta A. (2011). Chronic suppurative otitis media in children of Luanda. *Angola. Acta Paediatr.* 100 e84–e88. 10.1111/j.1651-2227.2011.02192.x 21477130

[B65] Truong D. Copeland J. W. Brumell J. H. (2014). Bacterial subversion of host cytoskeletal machinery: hijacking formins and the Arp2/3 complex. *Bioessays* 36 687–696. 10.1002/bies.201400038 24849003

[B66] Val S. Mubeen H. Tomney A. Chen S. Preciado D. (2015). Impact of *Staphylococcus epidermidis* lysates on middle ear epithelial proinflammatory and mucogenic response. *J. Investig. Med.* 63 258–266. 10.1097/JIM.0000000000000127 25503091

[B67] Verhoeff M. van der Veen E. L. Rovers M. M. Sanders E. A. Schilder A. G. (2006). Chronic suppurative otitis media: a review. *Int. J. Pediatr. Otorhinolaryngol.* 70 1–12. 10.1016/j.ijporl.2005.08.021 16198004

[B68] Wang Y. Zhou H. Wu B. Zhou Q. Cui D. Wang L. (2015). PKC Isoforms distinctly regulate propofol-induced endothelium-dependent and endothelium-independent vasodilation. *J. Cardiovasc. Pharmacol.* 66 276–284. 10.1097/FJC.0000000000000275 25970840

[B69] Wanger T. M. Dewitt S. Collins A. Maitland N. J. Poghosyan Z. Knäuper V. (2015). Differential regulation of TROP2 release by PKC isoforms through vesicles and ADAM17. *Cell. Signal.* 27 1325–1335. 10.1016/j.cellsig.2015.03.017 25817572

[B70] Woo J. I. Kil S. H. Brough D. E. Lee Y. J. Lim D. J. Moon S. K. (2015). Therapeutic potential of adenovirus-mediated delivery of β-defensin 2 for experimental otitis media. *Innate Immun.* 21 215–224. 10.1177/1753425914534002 24842664

[B71] Woo J. I. Kil S. H. Pan H. Lee Y. J. Lim D. J. Moon S. K. (2014). Distal NF-kB binding motif functions as an enhancer for nontypeable *H. influenzae*-induced DEFB4 regulation in epithelial cells. *Biochem. Biophys. Res. Commun.* 443 1035–1040. 10.1016/j.bbrc.2013.12.091 24368180 PMC3940165

[B72] Woodruff W. A. Hancock R. E. (1989). *Pseudomonas aeruginosa* outer membrane protein F: structural role and relationship to the *Escherichia coli* OmpA protein. *J. Bacteriol.* 171 3304–3309. 2498289 10.1128/jb.171.6.3304-3309.1989 PMC210050

[B73] Xiao Y. Yao Y. Jiang H. Lu C. Zeng S. Yu L. (2015). Regulation of uridine diphosphate-glucuronosyltransferase 1A3 activity by protein phosphorylation. *Biopharm. Drug Dispos.* 36 520–528. 10.1002/bdd.1963 26094731

[B74] Yakhnina A. A. McManus H. R. Bernhardt T. G. (2015). The cell wall amidase AmiB is essential for *Pseudomonas aeruginosa* cell division, drug resistance and viability. *Mol. Microbiol.* 97 957–973. 10.1111/mmi.13077 26032134 PMC4646093

[B75] Yeo S. G. Park D. C. Hong S. M. Cha C. I. Kim M. G. (2007). Bacteriology of chronic suppurative otitis media-a multicentre study. *Acta Otolaryngol.* 127 1062–1067. 10.1080/00016480601126978 17851935

