# Supplementary material for: Pseudomonas aeruginosa Activates PKC-Alpha to Invade Middle Ear Epithelial Cells
Source: Front Microbiol. 2016 Mar 4;7:255. doi: 10.3389/fmicb.2016.00255 (PMC4777741; doi:10.3389/fmicb.2016.00255)
Supplement: Supplementary file 1 [file Data_Sheet_1.PDF]

Supplementary Figure Legends

Supplementary Figure 1: Chelerythrine significantly prevents the invasion of *P. aeruginosa* inside HMEECs. Cells were pretreated with different concentrations of Chelerythrine and then infected with *P. aeruginosa*. The adhesion and invasion of bacteria was then determined. The results are expressed as percentage compared to the bacterial adhesion/invasion in untreated infected cells. Data represents mean ± SD and is representative of four individual experiments carried out in triplicate. *P < 0.01 or **P < 0.001 compared to control.

Supplementary Figure 2: Effect of Calphostin C on invasion of HMEECs by *P. aeruginosa*. Cells pretreated with different concentrations of Calphostin C were infected with *P. aeruginosa*. The adhesion and invasion of bacteria was then determined. The results are expressed as percentage compared to the bacterial adhesion/invasion in untreated infected cells. Data represents mean ± SD and is representative of four individual experiments carried out in triplicate. *P < 0.01 or **P < 0.001 compared to control.
