# Supplementary figures and images for: Pseudomonas aeruginosa Activates PKC-Alpha to Invade Middle Ear Epithelial Cells
Source: Front Microbiol. 2016 Mar 4;7:255. doi: 10.3389/fmicb.2016.00255 (PMC4777741; doi:10.3389/fmicb.2016.00255)

Supplementary Figure 1

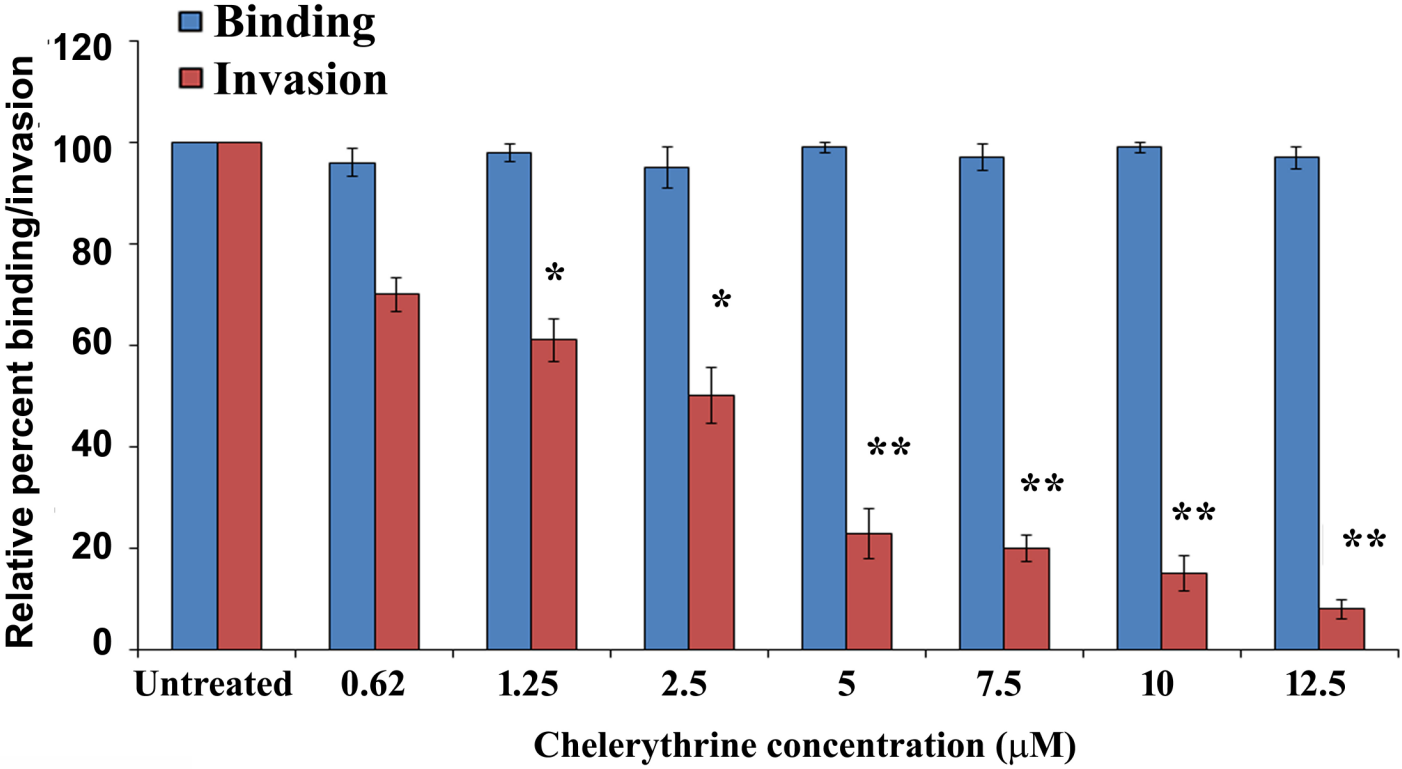

**Supplementary Figure 2**

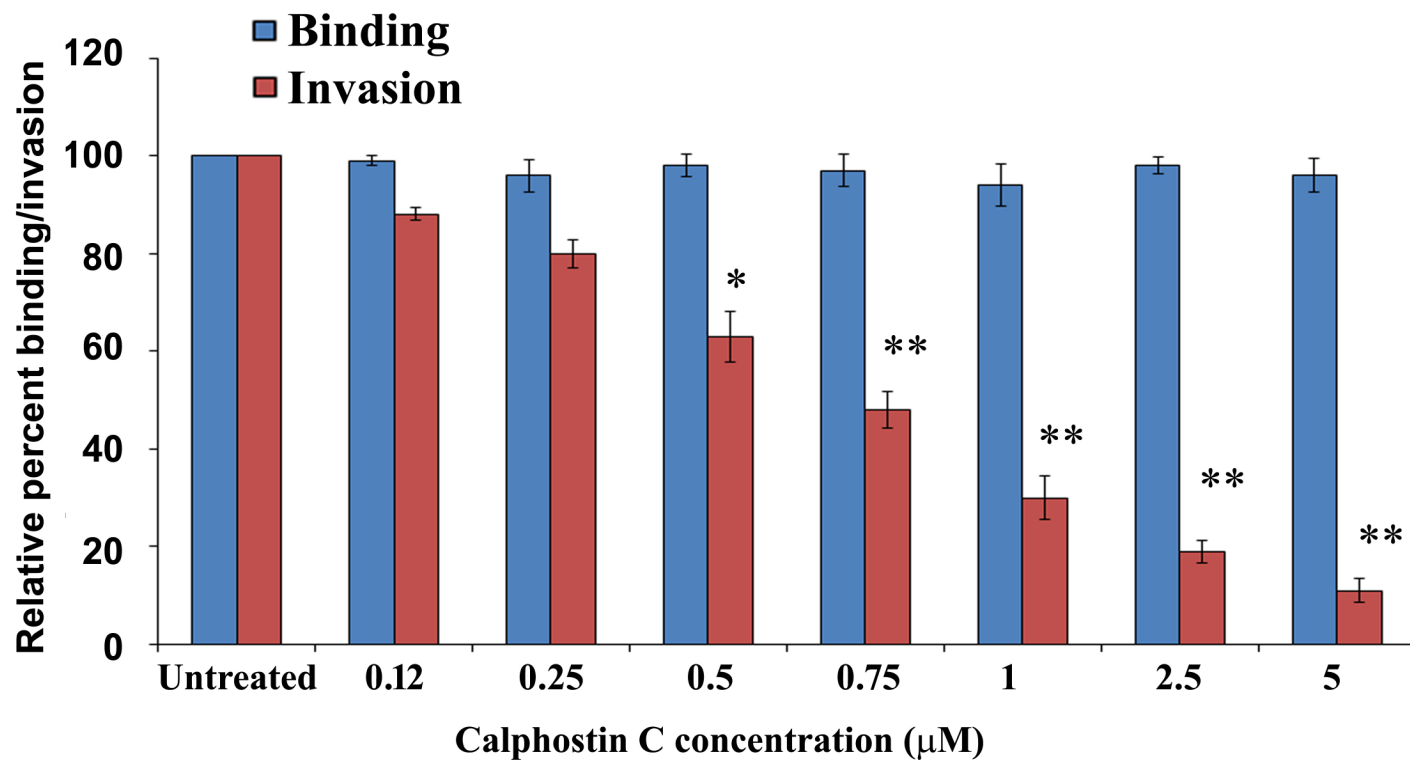

Supplement: Supplementary file 2 [file Image_1.PDF]
